# Intracellular IFN-γ and IL-4 levels of CD4 + and CD8 + T cells in the peripheral blood of naturally infected (*Leishmania infantum*) symptomatic dogs before and following a 4-week treatment with miltefosine and allopurinol: a double-blinded, controlled and cross-sectional study

**DOI:** 10.1186/s13028-023-00666-1

**Published:** 2023-01-26

**Authors:** Dimitrios T. Matralis, Alexander F. Koutinas, Ioanna E. Papadogiannaki, Elias G. Papadopoulos, Emmanouil I. Papadogiannakis

**Affiliations:** 1 Vets4life Veterinary Clinic, 24 Marathonos Av., Pikermi, 190 09 Athens, Greece; 2 Quality Veterinary Practice, 90 Kapodistriou Str., 383 33 Volos, Greece; 3 Small Animal Dermatology Clinic, 2 Krystalli Str., Alimos, 174 55 Athens, Greece; 4 Laboratory of Parasitology and Parasitic Diseases, School of Veterinary Medicine, University Campus, Aristotle University of Thessaloniki, 541 24 Thessaloniki, Greece; 5 School of Public Health, University of West Attica, 196 Alexandras Av., 115 21 Athens, Greece

**Keywords:** Canine leishmaniosis, CD4 + cells, CD8 + cells, Intracellular cytokines, Treatment

## Abstract

**Background:**

Canine leishmaniosis (CanL) is a systemic disease caused by the protozoan parasite *Leishmania infantum* with a wide spectrum of clinical signs, with cutaneous, ocular, renal and lymphoreactive conditions prevailing in the clinical setting. The immune system plays a pivotal role in the evolution of *Leishmania* infection and its response to antileishmanial treatment. Cytokines are important immune response mediators that are released by activated lymphocytes and less so by other immunocytes. In dogs with leishmaniosis, IFN-γ and IL-4 have been recognized as the main activators of cellular and humoral immunity, respectively. The objective of this study was to investigate intracellular IL-4 and IFN-γ expression by CD4 + and CD8 + lymphocytes in the peripheral blood of symptomatic dogs before and after combined antileishmanial treatment with miltefosine and allopurinol.

**Results:**

Postantileishmanial treatment CD4 + IL-4 + and CD8 + IL-4 + cell counts were significantly decreased, although no similar changes were observed in the comparisons made between the pre- and posttreatment CD4 + IFN-γ + and CD8 + IFN-γ + counts and ratios.

**Conclusion:**

The findings indicate that IL-4 production by T cells may facilitate the symptomatic phase of CanL, whereas IFN-γ production by CD4 + and CD8 + cells may indicate its negligible role in the evolution of natural CanL and perhaps the equivocal positive influence of antileishmanial treatment.

## Background

Canine leishmaniosis (CanL) is a systemic disease caused by the protozoan parasite *Leishmania infantum* and transmitted by female phlebotomine sand flies in Mediterranean countries and elsewhere in the world [1–3]. The manifestations of CanL are a wide spectrum of clinical signs originating from multiple organs, with cutaneous, ocular, renal and lymphoreactive conditions prevailing in the clinical setting [2, 4, 5].

The immune system plays a pivotal role in the evolution of *Leishmania* infection and its response to antileishmanial treatment [6], but the cellular components remain only partially elucidated, although it has been recognized that the underlying immunopathogenicity is mainly of the Th1 type [7, 8]. However, in this disease, the number of CD4 + T cells and the CD4 + /CD8 + ratio are decreased, thus permitting the multiplication and spread of the parasite [9–11]. Cytokines are important immune response mediators released by activated lymphocytes and less so by other immunocytes. In dogs with leishmaniosis, IFN-γ and IL-4 have been recognized as the main activators of cellular and humoral immunity, respectively [12–15].

A positive correlation between high IL-4 levels and clinical disease appears to prevail in a murine model of visceral leishmaniosis, but no such immune dichotomy has been observed in CanL [16]. Data from previous studies on CanL have indicated that the altered homoeostasis found between CD4 + and CD8 + lymphocyte subsets is most likely the consequence of CanL itself, at least in symptomatic dogs [16–18]. Indeed, the numbers of CD4 + IL-4 + and CD8 + IL-4 + lymphocytes were significantly higher in the peripheral blood of symptomatic dogs than in that of asymptomatic or parasitologically negative dogs, thus emphasizing the role that these cells may play in shaping humoral immunity and most likely in the progression of CanL with the advent of IL-4 release [16].

The aim of this study was to investigate intracellular IL-4 and IFN-γ expression by CD4 + and CD8 + lymphocytes in the peripheral blood of symptomatic dogs before and after treatment with miltefosine and allopurinol in an attempt to further elucidate the immune response at an intracellular level.

## Methods

### Study population, selection criteria and study design

The study population comprised 10 symptomatic dogs with *L. infantum* infection diagnosed by a combination of lymph node cytology, indirect fluorescent antibody (IFA) serology of peripheral blood, and PCR (Table 1). These animals were subsequently treated with miltefosine (2 mg/kg PO, once daily) and allopurinol (10 mg/kg PO, twice daily) for four weeks. Blood samples were taken from the diseased dogs before (“pretreatment”) and at the end of the 4-week treatment (“posttreatment”). Blood samples were also obtained from 10 breed-, sex- and age-matched but clinically healthy dogs originating from the same geographic area that tested negative for *L. infantum* infection according to IFA serology of peripheral blood and PCR; these dogs comprised the control group (“controls”). All 20 dogs resided in the metropolitan area of Athens, Greece, and were included in the study by having met the following criteria: (1) a status of being currently vaccinated except for antileishmanial vaccination, (2) annual deworming prevention, (3) no subjection to antileishmanial treatment for at least 1 year before the initiation of the study, (4) seronegativity to other locally common vector-borne infectious diseases (ehrlichiosis, anaplasmosis, babesiosis, dirofilariasis) and (5) the absence of clinical and/or laboratory evidence of other infectious or common diseases. Advice was informally requested from the ‘Bioethics Committee of the School of Public Health, University of West Attica’, although all blood samples used in the study had been collected as part of the routine procedure to diagnose CanL and monitor its treatment.

**Table 1:** Characteristics of the dogs

Dogs	Breed	Gender	Age (years)	Body weight (Kg)
1	Mixed	♀	3	16
2	German Shepherd	♂	4	39
3	Mixed	♂	5	21
4	Mixed	♂	4	24
5	Mixed	♀	4	22
6	Mixed	♀	3	23
7	Pit bull	♂	4	29
8	Mixed	♀	4	25
9	Mixed	♂	5	18
10	Mixed	♂	4	17

### Flow cytometry

Peripheral blood (5 mL), collected in lithium heparin vials, was transported within 1–3 h to the School of Public Health, University of West Attica, Athens, Greece. Flow cytometry analysis was performed as previously described [16, 19]. Cytokine production is one of the first steps of the immune response and can provide important information regarding the nature of any immunological response. As resting immune cells produce only a minimal amount of cytokines to meet their basic cellular requirements, cytokine profiles from inactivated blood might not accurately reflect the immune function status. Stimulation is necessary for the measurement of cytokine production. In this study, PMA and LSA were used as nonspecific and specific immunostimulants, respectively. Briefly, blood aliquots of 0.5 mL were added into plastic tubes with 0.5 mL RPMI 1640 culture medium. Monensin (MN) was added to all cell cultures at 3 μmol/L to prevent the release of cytokines from the cells. MN is derived from *Streptomyces cinnamonensis* and is a Na^+^ ionophore that disrupts intracellular Na^+^ and H^+^ gradients, exerting its greatest effects on the regions of the Golgi apparatus that are associated with the final stages of secretory vesicle maturation [20]. For the in vitro activation of CD4 + and CD8 + lymphocytes, phorbol-12-myristate-13-acetate (PMA) was added at a final concentration of 10 ng/mL, as well as ionomycin (Applichem, Darmstadt, Germany) at 2 μmol/L plus the Leishmania Soluble Antigen (LSA, at 10 μg/mL), which was kindly provided by the Pasteur Institute of Athens, Greece. Tubes were subsequently incubated at 37 °C in a humid 5% CO_2_ atmosphere for 4–6 h. Monoclonal antibodies specific for canine CD4 and CD8 cell surface antigens (rat anti-canine CD4:FITC, Subclass IgG2a, Cat. Number MCA 1038F, Serotec, Kidlington, Oxford, U.K. and rat anti-canine CD8:FITC, Subclass IgG1, Cat. Number MCA 1039F, Serotec, Kidlington, Oxford, U.K.) were added along with appropriate isotypic negative (rat IgG2a: FITC and rat IgG1: FITC, Serotec, Kidlington, Oxford, UK) and positive (mouse anti-human CD69: RPE, Beckman Coulter, Miami, FL, USA) controls and incubated at room temperature for 15 min. Cells were permeabilized by Leucoperm reagents A and B (Serotec, Kidlington, Oxford, UK), as previously described [16]. After the fixation and washing of cells, appropriate monoclonal antibodies specific for bovine IFN-γ and IL-4 along with appropriate isotypic negative controls (mouse IgG2a:RPE and mouse IgG1: RPE) (Serotec, Kidlington, Oxford, UK) at a concentration of 100 μg/mL were added and incubated at room temperature for 20 min. The above bovine monoclonal antibodies cross-reacted with canine cytokines. Samples were subsequently washed twice with PBS and analysed in a Coulter Epics-XL-MCL 4-colour cytometer with attached WINLIST Version 3.0 software (Verity, Topsham, ME, USA). The results were expressed as the percentage of CD4 + and CD8 + and cytokine-positive CD4 + and CD8 + cells counted in the gating area of lymphocytes.

### Statistical analyses

Pre- versus poststimulation data were compared by paired t tests. Other comparisons were made by ANOVA with repeated measures. The SPSS v. 20.0 (SPSS Inc., Chicago, IL, USA) software programme was employed for statistical analysis. Differences with calculated P values of < 0.05 were considered significant for all the comparisons made.

## Results

Compared to controls, the percentage of CD4 + T-lymphocytes in pretreatment dogs was found to be decreased at the resting phase as well as after LSA or PMA/iono immunostimulation (Table 2). Additionally, in posttreatment dogs, the lymphocytic counts were found to be increased significantly (P = 0.01) at the resting phase and after PMA but not after LSA immunostimulation (Table 2).

**Table 2:** Mean percentage (%) and standard deviation (SD) of CD4 + and CD8 + cells and their ratio before and after antileishmanial treatment at resting phase and following LSA or PMA/iono immunostimulation

	CD4 + cells			CD8 + cells			CD4 + /CD8 + ratio		
	Before treatment	After treatment	Controls	Before treatment	After treatment	Controls	Before treatment	After treatment	Controls
Resting phase	30.28 (1.42)	35.83 (1.61)	41.74 (5.88)	26.64 (2.13)	18.59 (1.38)	16.39 (2.90)	1.14 (0.13)	1.91 (0.07)	2.58 (0.42)
PMA/iono immunostimulation	23.79 (8.49)	30.59 (1.15)	35.94 (5.50)	18.09 (0.72)	15.46 (0.55)	11.99 (2.13)	1.46 (0.06)	1.97 (0.06)	3.03 (0.39)
LSA immunostimulation	29.83 (0.60)	35.49 (1.66)	38.37 (5.51)	26.50 (1.40)	19.08 (0.88)	16.43 (3.13)	1.12 (0.04)	1.86 (0.13)	2.4 (0.52)

The number of CD8 + T-lymphocytes was also found to be increased in symptomatic pretreatment dogs at both the resting phase and after immunostimulation by either LSA or PMA/iono (Table 2). Antileishmanial treatment resulted in a decrease in both at the resting phase and after LSA or PMA/iono immunostimulation, but the counts did not show a tendency to fully normalize (i.e., to control levels).

Compared to the controls, pretreatment CD4 + /CD8 + ratio was lower at both the resting and immunostimulation phases (LSA, PMA/iono), and although these values were increased posttreatment, there was no tendency towards full normalization in either instance (P < 0.001) (Table 2).

Pretreatment mean numbers of CD4 + IL-4 + T-lymphocytes were increased compared to those of the controls at the resting phase and immunostimulation phases of the study (P < 0.001) (Table 3), but posttreatment, these numbers decreased with both types of immunostimulation but did not show a tendency to approach the control group values (Table 3).

**Table 3:** Mean percentage (%) and standard deviation (SD) of CD4 + IL-4 +, CD8 + IL-4 +, CD4 + IFN-γ +, and CD8 + IFN-γ + cells before and after antileishmanial treatment at resting phase and following LSA or PMA/iono immunostimulation

	CD4 + IL-4 + cells			CD8 + IL-4 + cells			CD4 + IFN-γ + cells			CD8 + IFN-γ + cells		
	Before treatment	After treatment	Controls	Before treatment	After treatment	Controls	Before treatment	After treatment	Controls	Before treatment	After treatment	Controls
Resting phase	1.39 (0.21)	1.27 (0.18)	0.55 (0.15)	2.64 (1.10)	1.76 (0.38)	0.77 (0.26)	1.51 (0.32)	1.54 (0.31)	0.68 (0.20)	1.36 (0.51)	1.64 (0.19)	0.91 (0.21)
PMA/iono immunostimulation	1.91 (0.62)	1.29 (0.29)	0.64 (0.17)	2.82 (1.37)	1.74 (0.58)	0.76 (0.24)	3.91 (1.05)	4.16 (1.01)	2.27 (0.90)	5.63 (1.55)	5.66 (1.48)	1.80 (0.76)
LSA immunostimulation	3.34 (1.70)	1.55 (0.38)	0.60 (0.14)	5.08 (1.81)	2.03 (0.42)	0.67 (0.32)	3.49 (1.26)	3.02 (0.83)	0.58 (0.20)	4.65 (1.37)	3.86 (0.67)	0.59 (0.13)

Pretreatment CD8 + IL-4 + cell counts were similar to CD4 + IL-4 + cell counts, but following treatment, these counts decreased significantly (P = 0.001), both at the resting and poststimulation phases, although they did not reach the control counts (P < 0.05) (Table 3).

Pretreatment CD4 + IFN-γ + T-lymphocyte counts were increased at the resting and LSA or PMA/iono immunostimulation phases (P = 0.01), but these counts were not influenced by the treatment in either instance and remained higher than control counts. Similar observations were made regarding the CD8 + IFN-γ + T-lymphocyte counts (Table 3).

## Discussion

In this cross-sectional study, the mean percentages of CD4 + and CD8 + cells in clinically normal dogs (controls) at the resting phase were 40% and 17%, respectively (Table 2), similar to what has previously been reported in in vitro studies [9]. The mild decrease in their numbers following LSA or PMA/iono immunostimulation in posttreatment dogs and controls might be attributed to cellular apoptosis, which is known to occur within 4 to 6 h after immunostimulation [21], although a downregulation of the C molecule by PMA could not be excluded.

Compared with control counts, CD4 + cell counts were decreased before the start of antileishmanial treatment, but afterwards, by applying PMA/iono immunostimulation, they were increased, although they were still below the normal range. These results are in line with those of previous studies, indicating that along with CanL progression, the initial antigen-specific CD4 + T lymphocytic hyperreactivity eventually subsides [9]. Additionally, it is known that symptomatic CanL correlates with a substantial decrease in antigen-specific lymphocytic responsiveness [24]. The increase in CD4 + T-lymphocyte counts with the aid of both antileishmanial treatment and LSA immunostimulation could be explained by the activation of their previously anergic counterparts [25]. The opposite seems to be true for CD8 + cells, as their numbers have been found to decrease, although they do not show a tendency to fully normalize with antileishmanial treatment. The same changes have been noticed in the CD4 + /CD8 + ratio of symptomatic dogs, thus leading to the explanation of the relapses noticed after the end of the treatment in CanL patients [18].

In the controls, no significant differences could be noticed between CD8 + IL-4 + lymphocytes either after PMA/iono or LSA immunostimulation, in contrast to what was witnessed in CD4 + IL-4 + lymphocytes (Table 3), leading to the assumption that the main source of IL-4 secretion is the CD4 + lymphocyte subset, although other cell types were not investigated for IL-4 production. In human and murine models of visceral leishmaniasis, IL-4 is hardly detectable in CD4 + and CD8 + lymphocytes [26], which does not agree with canine findings, at least according to the results of this study. The increased numbers of CD4 + IFN-γ + and CD8 + IFN-γ + lymphocytes in the controls after nonspecific immunostimulation most likely imply IFN-γ secretion by both lymphocytic subpopulations. Each stimulant induces the production of a unique cytokine profile and weakly promotes the production of other cytokines. The PMA/ionomycin combination significantly increases the production of IFN-γ as opposed to IL-4. On the other hand, the nonstimulation of lymphocytes by LSA is not surprising, since LSA stimulates only cells previously sensitized by *Leishmania* antigens [22, 23].

It is interesting to note that before antileishmanial treatment, the comparison between lymphocyte subsets in terms of IL-4 levels after LSA immunostimulation tended to indicate an ever-increasing number of CD8 + cells as a specific reaction to leishmanial antigens (Table 3). This does not seem to apply to IFN-γ, hence justifying the characterization of CD8 + cells as non-IFN-γ inducers known to be associated with the advanced stages of the disease [24, 27]. As CanL progresses from subclinical to clinical, an impairment of specific CD4 + T-cell proliferation and IFN-γ production has been documented despite the differences regarding the substrate for cytokine measurements (blood plasma) and the nonspecific stimulant (ConA) applied [8, 24, 27]. Nevertheless, the cellular basis and mechanisms of antigen T-cell unresponsiveness in natural CanL have not been fully elucidated [27], and the combination of our data with those of other investigators [28, 29] leading to a positive correlation between increased IL-4 levels and clinical disease does not fully support the clear dichotomy of the immune response witnessed in the murine model of visceral leishmaniosis in the corresponding canine disease. Symptomatic canine leishmaniosis patients exhibit a mixed immune profile with a concomitant presence of increased IL-4- and IFN-γ-producing T cells and neutrophils, making them unable to control parasitic replication [8].

Antileishmanial treatment led to a decrease in CD4 + IL-4 + and CD8 + IL-4 + cells, while CD4 + IFN-γ + and CD8 + IFN-γ + cells remained unchanged (Table 3), thus correlating even indirectly IL-4 production with symptomatic disease and the concomitant increase in the parasitic load [29]. A limitation of the study is the restricted treatment time of only 28 days, and the dogs were not followed up for a long period of time. The possibility of alterations in the cytokine profile after prolonged treatment cannot be excluded. Early expression of IL-4, as measured in canine spleen cells, seems to play an important role in the prolongation of parasite persistence and parasitaemia despite the high expression of IFN-γ [28], and the fact that CD4 + IL-4 + and CD8 + IL-4 + numbers do not show a tendency to fully normalize with antileishmanial treatment may explain, even partially, the so often witnessed relapse of the clinical disease.

## Conclusions

The pretreatment CD4 + /CD8 + ratio was lower at both the resting and immunostimulation phases, and although these values were increased posttreatment, there was no tendency towards full normalization. Posttreatment CD4 + IL-4 + and CD8 + IL-4 + cell counts were decreased significantly, but no similar changes were observed in the comparisons made between the pre- and posttreatment CD4 + IFN-γ + and CD8 + IFN-γ + counts and ratios. It is likely that IL-4 production by T cells facilitates the symptomatic phase of CanL, whereas IFN-γ production by CD4 + and CD8 + cells may indicate its negligible role in the evolution of natural CanL as well as the equivocal influence of antileishmanial treatment.

## References

[1] Solano-Gallego L, Miró G, Koutinas A, Cardoso L, Pennisi MG, Ferrer L, The LeishVet Group (2011). LeishVet guidelines for the practical management of canine leishmaniosis. Parasit Vectors.

[2] Solano-Gallego L, Koutinas A, Miró G, Cardoso L, Pennisi MG, Ferrer L (2009). Directions for the diagnosis, clinical staging, treatment and prevention of canine leishmaniosis. Vet Parasitol.

[3] Ciaramella P, Oliva G, De Luna R, Gradoni R, Ambrosio R, Cortese L (1997). A retrospective clinical study of canine leishmaniasis in 150 dogs naturally infected by *Leishmania infantum*. Vet Rec.

[4] Baneth G, Koutinas AF, Solano-Gallego L, Bourdeau P, Ferrer L (2008). Canine leishmaniosis-new concepts and insights on an expanding zoonosis: part one. Trends Parasitol.

[5] Koutinas AF, Polizopoulou ZS, Saridomichelakis MN, Argyriadis D, Fytianou A, Plevraki KG (1999). Clinical considerations on canine visceral leishmaniasis in Greece: a retrospective study of 158 spontaneous cases (1989–1996). J Am Anim Hosp Assoc.

[6] Giunchetti RC, Silveira P, Resende LA, Leite JC, Melo-Júnior OAO, Rodrigues-Alves ML (2019). Canine visceral leishmaniasis biomarkers and their employment in vaccines. Vet Parasitol.

[7] Chamizo C, Moreno J, Alvar J (2005). Semiquantitative analysis of cytokine expression in asymptomatic canine leishmaniasis. Vet Immunol Immunopathol.

[8] de Almeida Leal GG, Roatt BM, de Oliveira Aguiar-Soares RD, Carneiro CM, Giunchetti RC (2014). Immunological profile of resistance and susceptibility in naturally infected dogs by *Leishmania infantum*. Vet Parasitol.

[9] Papadogiannakis E, Andritsos G, Kontos V, Spanakos G, Koutis C, Velonakis E (2010). Determination of CD4+ and CD8+ T cells in the peripheral blood of dogs with leishmaniosis before and after prolonged allopurinol monotherapy. Vet J.

[10] Moreno J, Nieto J, Chamizo C, Gonzalez F, Blanco F, Barker D (1999). The immune response and PBMC subsets in canine visceral leishmaniasis before, and after, chemotherapy. Vet Immunol Immunopathol.

[11] Guerra LL, Teixeira-Carvalho A, Giunchetti RC, Martins-Filho OA, Reis AB, Corrêa-Oliveira R (2009). Evaluation of the influence of tissue parasite density on hematological and phenotypic cellular parameters of circulating leukocytes and splenocytes during ongoing canine visceral leishmaniasis. Parasitol Res.

[12] Pinelli E, Killick-Kendrick R, Wagenaar J, Bernadina W, del Real G, Ruitenberg J (1994). Cellular and humoral immune responses in dogs experimentally and naturally infected with *Leishmania infantum*. Infect Immun.

[13] Day M. Implications of the immune system during infection by *Leishmania* organism in canine. Proceedings of International Congress on Canine Leishmaniosis. Naples, Italy 2004;21–8.

[14] Lage RS, Oliveira GC, Busek SU, Guerra LL, Giunchetti RC, Corrêa-Oliveira R (2007). Analysis of the cytokine profile in spleen cells from dogs naturally infected by *Leishmania chagasi*. Vet Immunol Immunopathol.

[15] Quinnell RJ, Courtenay O, Shaw MA, Day MJ, Garcez LM, Dye C (2001). Tissue cytokine responses in canine visceral leishmaniasis. J Infect Dis.

[16] Matralis D, Papadogiannakis E, Kontos V, Papadopoulos E, Ktenas E, Koutinas A (2016). Detection of intracellular IFN-γ and IL-4 cytokines in CD4+ and CD8+ T cells in the peripheral blood of dogs naturally infected with *L. infantum*. Parasite Immunol.

[17] Papadogiannakis EI, Koutinas AF, Saridomichelakis MN, Vlemmas J, Lekkas S, Karameris A (2005). Cellular immunophenotyping of exfoliative dermatitis in canine leishmaniosis (*Leishmania infantum*). Vet Immunol Immunopathol.

[18] Papadogiannakis E. Contribution to the study of immunopathogenesis of exfoliative dermatitis in canine leishmaniosis (*Leishmania infantum*) [PhD thesis]. 2003; Veterinary Faculty, University of Thessaloniki, Greece.

[19] Papadogiannakis EI, Kontos VI, Tamamidou M, Roumeliotou A (2009). Determination of intracellular cytokines IFN-γ and IL-4 in canine T-lymphocytes by flow cytometry following whole blood culture. Can J Vet Res.

[20] Mollenhauer HH, Morré DJ, Rowe LD (1990). Alteration of intracellular traffic by monensin; mechanism, specificity and relationship to toxicity. Biochim Biophys Acta.

[21] Sewell WA, North ME, Webster AD, Farrant J (1997). Determination of intracellular cytokines by flow-cytometry following whole-blood culture. J Immunol Methods.

[22] Ghersetich I, Menchini G, Teofoli P, Lotti T (1999). Immune response to *Leishmania* infection in human skin. Clin Dermatol.

[23] Janeway CA, Travers P, Walport M (2008). Immunobiology: the immune system in health and disease.

[24] Boggiatto PM, Ramer-Tait AE, Metz K, Kramer EE, Gibson-Corley K, Mullin K (2010). Immunologic indicators of clinical progression during canine *Leishmania infantum* infection. Clin Vaccine Immunol.

[25] Papadogiannakis EI, Koutinas AF (2015). Cutaneous immune mechanisms in canine leishmaniosis due to *Leishmania infantum*. Vet Immunol Immunopathol.

[26] Pala P, Hussell T, Openshaw P (2000). Flow cytometric measurement of intracellular cytokines. J Immunol Methods.

[27] Solano-Gallego L, Montserrat-Sangrà S, Ordeix L, Martínez-Orellana P (2016). *Leishmania infantum*-specific production of IFN-γ and IL-10 in stimulated blood from dogs with clinical leishmaniosis. Parasit Vectors.

[28] Strauss-Ayali D, Baneth G, Jaffe CL (2007). Splenic immune responses during canine visceral leishmaniasis. Vet Res.

[29] Alves CF, de Amorim IF, Moura EP, Ribeiro RR, Alves CF, Michalick MS (2009). Expression of IFN-gamma, TNF-alpha, IL-10 and TGF-beta in lymph nodes associates with parasite load and clinical form of disease in dogs naturally infected with *Leishmania chagasi*. Vet Immunol Immunopathol.

